# Optimization of Flexible Rotor for Ultrasonic Motor Based on Response Surface and Genetic Algorithm

**DOI:** 10.3390/mi16010054

**Published:** 2024-12-31

**Authors:** Bo Chen, Jiyue Yang, Haoyu Tang, Yahang Wu, Haoran Zhang

**Affiliations:** School of Intelligent Equipment, Shandong University of Science and Technology, Tai’an 271019, China

**Keywords:** ultrasonic motor rotor, kriging, MOGA, optimize the design

## Abstract

The flexible rotor, as a crucial component of the traveling wave rotary ultrasonic motor, effectively reduces radial friction. However, issues such as uneven contact between the stator and rotor, as well as rotor-deformation-induced stress, still persist. This paper presents an optimization method that combines the Kriging response surface model with a multi-objective genetic algorithm (MOGA). Drawing on the existing rotor structure, a novel rotor design is proposed to match the improved TRUM60 stator. During the optimization process, the contact surface between the stator and rotor is taken as the optimization target, and an objective function is established. The Kriging response surface model is constructed using Latin hypercube sampling, and an MOGA is employed to optimize this model, allowing the selection of the optimal balanced solution from multiple candidate designs. Following stator optimization, the objective function value decreased from 0.631 to 0.036, and the maximum contact stress on the rotor inner ring was reduced from 32.77 MPa to 9.96 MPa. Experimental validation confirmed the reliability of this design, significantly improving the overall performance and durability of the motor.

## 1. Introduction

A traveling wave rotary ultrasonic motor (TRUM) operates by exciting the stator to vibrate through the inverse piezoelectric effect applied to the piezoelectric material. This vibration drives the rotor to rotate within the ultrasonic frequency range by transferring energy through the friction between the stator and rotor [1]. TRUMs are characterized by their low speed and high torque, high precision [2], fast response [3], quiet operation, and immunity to electromagnetic interference. Due to these properties, TRUMs are extensively utilized in the medical, aerospace, and automotive industries [4,5].

Aspects such as structural design and parameters can have an impact on the performance of ultrasonic motors [6,7]. Recent studies have investigated various aspects of ultrasonic motors. Mustafa et al. [8] used the principle that the maximum driving efficiency is consistent with the minimum driving current, used the extreme value search technique to achieve stable tracking of the optimal efficiency, and changed the output power of the motor by improving the pre-pressure dynamic control system. Hu et al. [9] used Workbench to optimize the design of the four dimensions of the stator and proposed a flange-shaped stator designed to improve the amplitude value of the particle value of the stator end face. Xie et al. [10] proposed a stator structure of a miniature ultrasonic motor with a suspended annular stator in B15 mode through the study of the NEMS manufacturing process. Guo et al. [11] designed a spherical stator synchronously self-aligning structure ultrasonic motor and innovatively applied the automatic centering characteristics of a three-jaw chuck to the field of ultrasonic motors. Cai et al. [12] established a thermo-electrical-mechanical coupling analysis model with the stator and rotor as the research object and proposed the superposition of two layers of piezoelectric ceramic plates of equal thickness to increase the resonant frequency of the stator when the temperature rises. Duan et al. [13] designed a flexible rotor that operates at low pressure with ultra-low preload, significantly enhancing performance through experimental validation. Lv et al. [14,15] developed a dynamic wear assessment model based on wear theory to predict the amount of wear in response to temperature variations and to obtain the effect of the variation in temperature rise on the output performance of the motor. Wang et al. [16] proposed an asymmetric structure ultrasonic motor and conducted size design and prototype production. The performance improvement was verified through experiments. Pan et al. [17] investigated the relationship between preload and load torque for the TRUM-70 ultrasonic motor. A review of the above studies reveals that they all have been conducted on the pre-stressing, stator design and temperature rise of ultrasonic motors, ignoring the important role of the rotor in ultrasonic motors.

In the design of ultrasonic motors, employing a flexible rotor instead of a rigid one presents several advantages [18]. A flexible rotor not only reduces wear between the stator and rotor but also simplifies the mechanism design. It can enhance the amplitude ratio between the stator and rotor while decreasing radial pressure. Hagedorn et al. [19] explained the importance of flexible rotors in traveling wave ultrasonic motors. If the flexibility of the rotor is not taken into account, the torque and speed characteristics of the motor cannot be better measured. Chen et al. [20] explained the phenomenon that appropriate rotor damping can improve motor performance by establishing a rigid–flexible coupling model of flexible rotors, providing a theoretical basis for the structural design of flexible rotors. Ren et al. [18] proposed a scheme based on equivalent contact pressure to evaluate the performance of flexible rotors, revealing how structural parameters influence the motor’s output performance. Chen et al. [21,22] analyzed the impact of rotor elasticity on contact stress and suggested a mechanism for performance enhancement based on rotor flexibility. Existing studies primarily concentrate on the kinetic analysis or performance evaluation of flexible rotors, with limited attention given to optimization design. By designing a new flexible rotor model, the issue of friction between the stator and rotor can be effectively addressed. However, traditional methods exhibit certain limitations in determining the dimensions of the rotor model, resulting in suboptimal outcomes. The Kriging model and genetic algorithm each possess unique advantages when addressing data processing and optimization problems. By selecting the appropriate method based on the specific problem, one can achieve optimal solutions. Utilizing these approaches to optimize rotor structures can significantly enhance performance.

**Figure 1:**
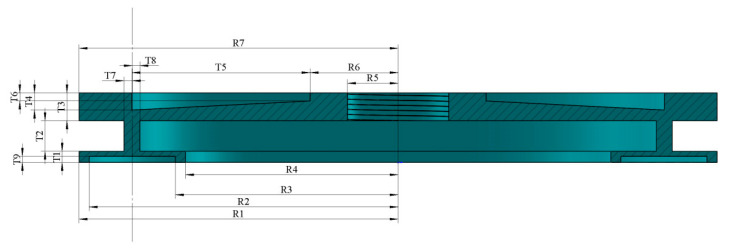
Cross-sectional view of the rotor structure.

This paper focuses on the optimization and design of a flexible rotor, using the traveling wave rotary ultrasonic motor (TRUM60) as the model. Improvements are made to the contact surface between the stator and rotor, drawing upon design and optimization methods from the relevant literature [23]. A new rotor structure is proposed, with the semi-section diagram and key parameters illustrated in Figure 1. The Kriging response surface model is developed using the Latin hypercube random sampling method, and a multi-objective genetic algorithm is employed to globally optimize the rotor design parameters. This approach aims to identify the optimal design solution.

## 2. Establishment of the Optimization Function

### 2.1. Establishment of Rotor Optimization Space

In ultrasonic motors, the rotor is in close contact with the stator, necessitating consideration of rotor deformation under these conditions [24]. The motor operates through high-frequency vibrations of the stator while the rotor rotates at a specific speed, resulting in complex interactions between the stator and rotor. Given the challenges associated with directly applying finite element analysis in such scenarios, this study focuses on optimizing rotor deformation under static conditions to streamline the design process.

The optimization process begins by defining the initial dimensions and design parameters of the rotor, based on established design guidelines and the structural characteristics of the TRUM60 ultrasonic motor, as presented in Table 1. Furthermore, the rotor dimensions must align with fixed parameters influenced by constraints related to the design of the contact surface, as outlined in Table 2. To further reduce rotor mass and cost, specific design considerations are implemented. The contact height between the rotor and stator is optimized to ensure complete coverage of the stator. The wall thickness of the contact surface is fixed at 1.5 mm, while the contact surface width is maintained at 8.5 mm. This width exceeds the difference between the inner and outer diameters of the stator teeth, thereby facilitating the installation process and mitigating potential machining errors associated with excessive tightness. These design strategies are employed to enhance performance and precision while ensuring cost-effectiveness in the rotor design.

**Figure 2:**
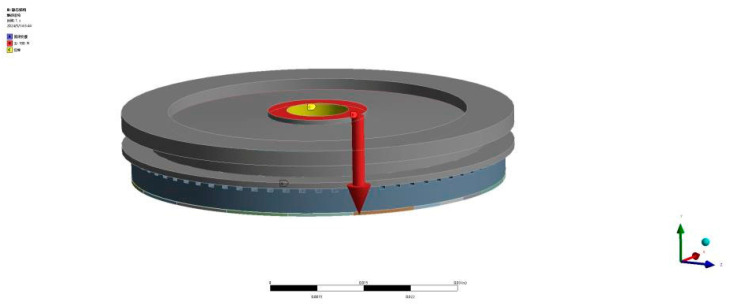
Constraint and boundary condition settings.

The rotor is fabricated from aluminum alloy, with PTFE chosen as the contact friction material between the stator and rotor. The relevant material properties are listed in Table 3. In the initial optimization process, the effect of preload is not considered, and a preliminary preload of 100 N is applied. The finite element model of the rotor, utilizing SOLID45 elements, is shown in Figure 2, where a vertical preload of 100 N is applied to the designated area. The finite element model includes specific constraints and boundary conditions. The contact surface between the stator tooth and the rotor’s friction layer is modeled as a frictional contact with a coefficient of 0.15, while other contact interfaces are treated as bonded. Fixed constraints are applied to the three bolt hole locations in the stator’s elastic matrix, and radial displacement constraints are imposed on the rotor web, with the axial direction remaining unconstrained. This setup allows for static analysis of the rotor.

Due to the nonlinear contact characteristics between the rotor and stator, accurately measuring displacements at each point on the contact surface is challenging [25,26]. To simplify the finite element analysis of the rotor, the contact surface is modeled as a fixed support. This approach enables the analysis of stresses on both the inner and outer sides of the contact interface during rotor deformation. By comparing the stress distribution between these two sides, it is possible to evaluate the uniformity of the contact between the rotor and stator.

### 2.2. Constructing the Rotor Optimization Function

The primary focus of this paper is the multi-objective optimization problem associated with flexible rotors, aimed at achieving uniform contact between the stator and rotor friction surfaces while minimizing contact stress. The design space for the initial dimensions and the allowable stress of the rotor are taken into account as constraints. Accordingly, Reference [27] established the following optimization model:

(1) $$\left\{\begin{array}{l} \min\left[M_{1}\left(x\right),M_{2}\left(x\right)\right]\\ x={\left(T1,T2,T3,T4,T5,T6,T7,T8,R6\right)}^{T}\\ M_{2}\left(x\right)\leq \sigma_{s} \end{array}\right.$$

In the equation, $M_{1}$ represents the polynomial objective function [27]. When the optimization goal $M_{1}$ is minimized, it indicates uniform contact between the stator and rotor. The parameters $\sigma_{s}$ correspond to the fixed dimensions listed in Table 2.

## 3. Specific Implementation Scheme

### 3.1. Latin Hypercube Sampling Method

To construct an accurate response surface model, it is essential to collect a sufficient and comprehensive set of data points, as the accuracy of the model depends on well-supported data [28]. To ensure that the data points are uniformly distributed and thoroughly cover the optimization space, this study employs the Latin hypercube sampling (LHS) method. LHS represents a significant advancement in sampling techniques, offering the advantage of accurately reconstructing the input data distribution with relatively few samples compared to other methods. The essence of LHS lies in stratified sampling, where the input probability distribution is divided into equal intervals within the range (0, 1) based on the cumulative probability curve. Sample points are then randomly selected within these stratified intervals, ensuring that representative values are chosen from each interval and effectively reconstructing the original probability distribution.

Latin hypercube sampling (LHS) is fundamentally based on the principle of stratified sampling rather than a specific formula. The primary objective is to ensure that each interval of the parameter space is equally and randomly represented, thereby preventing both oversampling and undersampling in any region. This method ensures more uniform and comprehensive coverage of the parameter space, which enhances the accuracy of the response surface model.

The sampling points obtained using the LHS method will be employed in finite element software for calculations. Data that do not satisfy the predefined optimization boundary conditions will be excluded, while the remaining valid data will be utilized to construct the response surface model. To balance the computational workload with the accuracy of model fitting, a final dataset of 147 design points is generated following the screening process.

### 3.2. Kriging Response Surface Modeling

The Kriging model, based on Kriging interpolation [29], is a powerful approach for modeling that not only provides estimates of unknown functions but also quantifies the associated errors. It is recognized as the best linear unbiased estimator for real computer models [30]. Kriging is a semiparametric interpolation technique that estimates unknown values using known data [31]. One of its primary advantages is its flexibility and adaptability to various spatial structures, achieved by adjusting the shape and range parameters of the semi-variance function. This adaptability makes Kriging a robust analytical and predictive tool for complex spatial data. Moreover, Kriging effectively addresses data trends and non-stationarity, positioning it as a preferred method for spatial data interpolation and prediction. Through its mathematical and statistical framework, Kriging response surface models can accurately fit each design point [32].

The principle of Kriging can be summarized in the following steps:

Structural Analysis: The analysis commences with the construction of the semi-variance function, which is derived from the examination of the spatial autocorrelation of the data. The semi-variance function elucidates the spatial continuity between samples by quantifying the relationship between the distance separating two sample points and the difference in their values. First, the semi-variance function $\gamma \left(h\right)$ is defined to quantify the correlation of spatial data, where h is the spatial distance. For any two locations $x_{i}$ and $x_{j}$, whose observations are $Z\left(x_{i}\right)$ and $Z\left(x_{j}\right)$, the semi-variance function is defined as follows:

(2) $$\gamma \left(h\right)=\frac{1}{2}Var\left[Z\left(x_{i}\right)-Z\left(x_{j}\right)\right]=\frac{1}{2N\left(h\right)}{\sum_{N\left(h\right)}\left[Z\left(x_{i}\right)-Z\left(x_{j}\right)\right]}^{2}$$

where $N\left(h\right)$ is the number of position pairs with a distance of h.

Model Fitting: In this step, a suitable theoretical model—such as spherical, exponential, or Gaussian ones—is selected to fit the empirical semi-variance function. This fitting process aims to accurately describe the spatial correlation between data points using mathematical formulations. By selecting an appropriate model, the theoretical semi-variance function can effectively represent the spatial structure observed in the empirical data, thus facilitating more precise interpolation and prediction.

Kriging Equation Solution: Using the selected semi-variance model, the Kriging equation is applied to estimate values at unknown locations. This approach takes into account the spatial coordinates and observed values of all known data points, as well as their spatial relationships with the unknown points. By solving a system of linear equations, the optimal weights are determined. The primary objective of Kriging is to minimize variance in the prediction error. The estimated value $\hat{Z}\left(x_{0}\right)$ at the unknown location $x_{0}$ is calculated as a weighted sum of the known values as follows:

(3) $$\hat{Z}\left(x_{0}\right)=\sum_{i=1}^{n}\lambda_{i}Z\left(x_{i}\right)$$

where $\lambda_{i}$ is the weighting factor that satisfies $\sum_{i=1}^{n}\lambda_{i}Z\left(x_{i}\right)$ to ensure no bias.

Error Estimation: Kriging provides an assessment of the uncertainty in the estimates for each prediction point, typically by calculating the prediction standard deviation. The variance in the prediction error, known as the Kriging variance, is given by the following:

(4) $$\sigma^{2}=Var\left[Z\left(x_{0}\right)-\hat{Z}\left(x_{0}\right)\right]=\sum_{i=1}^{n}\sum_{j=1}^{n}\lambda_{i}\lambda_{j}\gamma \left(xi,x_{j}\right)-2\sum_{i=1}^{n}\lambda_{i}\gamma \left(x_{i},x_{0}\right)+\gamma \left(0\right)$$

By solving the system of linear equations, the weights that minimize the Kriging variance can be determined.

Kriging Equations to Construct the System: The problem is transformed into the form of solving a system of linear equations, typically incorporating constraints introduced by the Lagrange multiplier method. This process ultimately results in a Kriging system defined as follows:

(5) $$\left\{\begin{array}{l} \sum_{j=1}^{n}\lambda_{i}\gamma \left(x_{i},x_{j}\right)+\mu =\gamma \left(x_{i},x_{0}\right)\;\mathrm{for}i=1,...,n\\ \sum_{i=1}^{n}\lambda_{i}=1 \end{array}\right.$$

where μ is the Lagrange coefficient, and by solving this system of linear equations, we can obtain the weights $\lambda_{i}$, and thus the predicted value of to the unknown position $\hat{Z}\left(x_{0}\right)$ and the variance in its prediction error $\sigma^{2}$.

**Figure 3:**
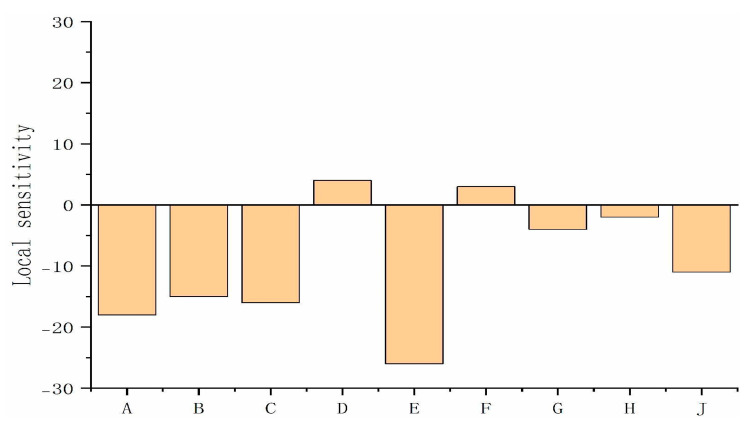
The local sensitivity of the objective function basis function.

In practical simulations, the response surface model can become highly intricate and expansive due to the multitude of parameters and the inclusion of cross, quadratic, and cubic terms. To address this complexity, the present study employs a strategic approach by selecting only those basis function terms that significantly influence the target variables. This focused selection not only streamlines the model but also enhances computational efficiency and clarity. By concentrating on the most impactful terms, this methodology ensures a more manageable and interpretable analysis, ultimately leading to more precise and actionable insights. The local sensitivity of the objective function basis function is shown in Figure 3 as follows: A—T1; B—T2; C—T3; D—T4; E—T5; F—T6; G—T7; H—T8; J—R6.

The final expression for the response surface model of the optimization function is formulated as follows:

M1 = 40.41 − 7.846 × A +
74.468 × B + 12.977 × C − 0.544 × D + 11.971 × E − 21.054 × F − 42.814 × G +
106.443 × H − 196.235 × J − 0.839 × AB + 1.881 × AC + 0.343 × AD + 0.549 × AE
+ 0.161 × AF − 1.280 × AG + 0.684 × AH − 4.040 × AJ − 19.348 × BC − 1.031 × BD
− 5.468 × BE − 5.292 × BF + 12.483 × BG − 22.338 × BH + 29.681 × BJ + 0.208 ×
CD − 0.764 × CE + 0.072 × CF − 7.403 × CG − 23.686 × CH − 13.737 × CJ − 0.514
× DE + 0.892 × DF − 1.333 × DG − 3.549 × DH − 4.669 × DJ + 0.144 × EF + 6.305
× EG + 3.041 × EH + 1.875 × EJ − 14.733 × FG − 17.088 × FH − 13.946 × FJ − 10.489
× GH + 83.473 × GJ + 69.455 × HJ + 0.138 × A2 − 5.659 × B2 + 6.725 × C2 +
0.173 × D2 − 2.017 × E2 + 1.112 × F2 + 14.648 × G2 − 15.451 × H2 + 108.745 × J

Equation A—R6; B—T6; C—T4; D—T5; E—T3; F—T2; G—T7; H—T8; J—T1.

**Figure 4:**
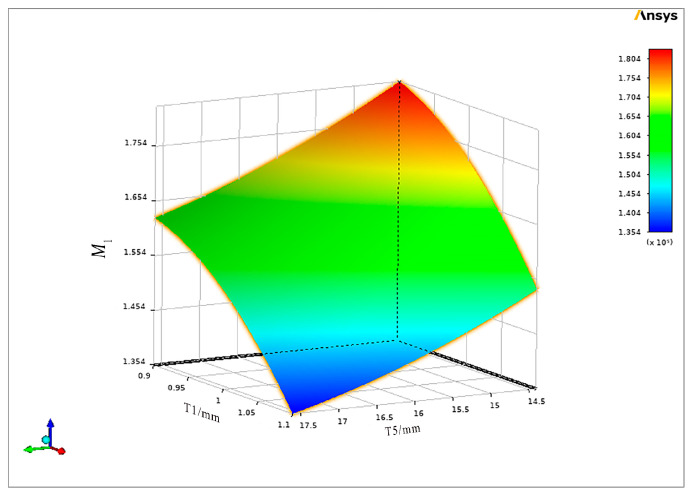
Response surface model diagram.

Further analysis reveals that the constructed response surface model exhibits robust predictive capability, with a coefficient of determination (R^2^) of 93.12%. This high R^2^ value indicates strong accuracy in the model’s predictions. Consequently, the response surface model effectively visualizes the interactions between significant parameters and their impacts on the optimization objective. The parameters that have the most influence on the optimization objective function are illustrated in Figure 4.

### 3.3. MOGA Optimized Response Surface

**Figure 5:**
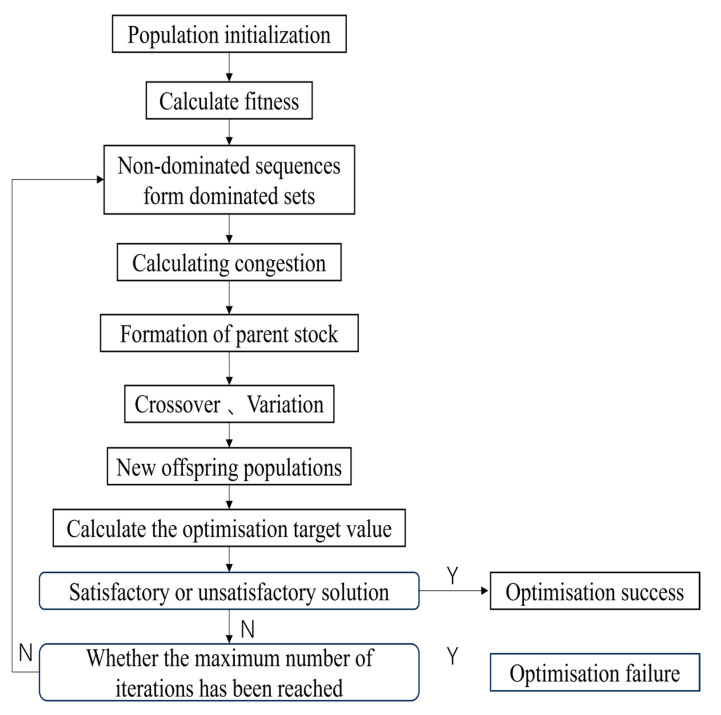
MOGA algorithm flow chart.

The multi-objective genetic algorithm (MOGA) is a robust approach for solving multi-objective optimization problems using genetic algorithms. The MOGA leverages the principles of natural selection to optimize solutions by employing mutation and crossover operations, thereby generating new populations that differ from their predecessors. This algorithm utilizes Pareto optimality to evaluate and select the best solutions, ensuring that all non-dominant solutions are considered with equal probability [33]. The optimization process of the MOGA is illustrated in the flowchart presented in Figure 5.

In the framework of multi-objective genetic algorithms (MOGAs), the optimization process begins by combining two parent chromosomes (referred to as “mother” chromosomes) through a crossover operation to generate a new chromosome, known as the “zygote”. This zygote inherits traits from both parents and has the potential to exhibit superior characteristics compared to those of its progenitors. Crossover occurs with a specified probability, producing two new chromosomes from each crossover point. Subsequently, the mutation process is applied, wherein one or more gene values within the chromosomes are modified. This introduces new genetic variations into the population, which may lead to improved solutions. Mutation is a critical component of the genetic algorithm, as it helps prevent premature convergence and avoids stagnation by exploring new regions of the solution space. The mutation is performed according to a predefined mutation probability, ensuring continued diversity within the population and enhancing the potential for finding optimal solutions.

The parameters used to implement the MOGA algorithm are listed in Table 4.

The optimization process is now complete. Given the large number of parameters and the high computational demands of the selected optimization technique, the workload was substantial. To efficiently address these challenges, the optimization was conducted using ANSYS Workbench 2022R1 and Design Expert software. This combination not only reduced the time required and minimized repetitive tasks but also ensured the stability and reliability of the optimization outcomes.

## 4. Experimental Results and Discussion

In accordance with the methodology outlined and the defined optimization objective function, an orthogonal experimental design was executed, yielding 147 sets of experimental data. A Kriging response surface model was subsequently developed based on these data points. The NSGA-II algorithm was then employed to numerically optimize the constructed response surface, resulting in three sets of optimal solutions, as summarized in Table 5.

**Figure 6:**
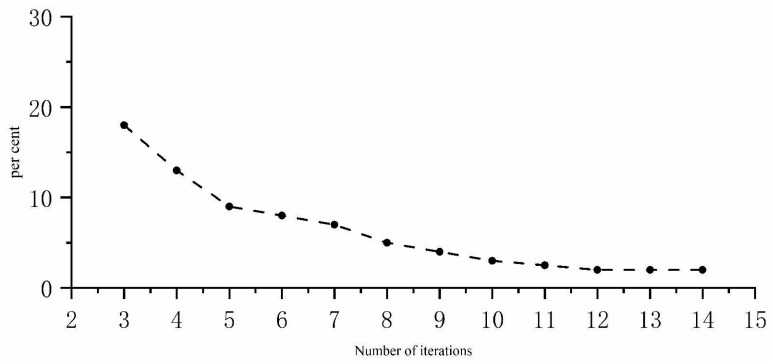
Optimize the iterative process of the objective function M1.

Figure 6 illustrates the progression of the primary optimization objective across iterations, demonstrating that convergence was achieved by the 12th iteration, which signifies the conclusion of the iterative process. The output values of the optimization objective function for the three candidate solutions are detailed in Table 6.

A lower value of M1 indicates improved uniformity of rotor contact and an extended operational lifespan. As presented in the table, candidate point 1 exhibits the minimum values for the optimization objective functions m1 and m2.

**Figure 7:**
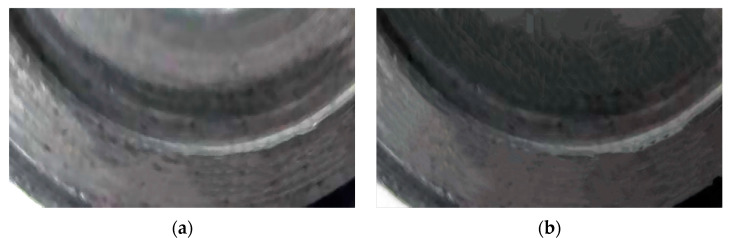
Wear before optimization (**a**) and wear after optimization (**b**).

To assess the effectiveness of the optimization results for M1, which quantifies the uniformity of contact between the stator and rotor—where a lower value indicates better uniformity—we conducted a validation experiment as follows: Initially, a standard flexible rotor was assembled into the motor. After operating the motor for a specified period, the wear pattern on the rotor was examined, as shown in Figure 7a. Subsequently, the rotor was manufactured according to the optimized dimensions outlined for Candidate 1 in Table 5 and reassembled into the motor. The motor was then subjected to extended performance testing, and the wear pattern of the optimized rotor was evaluated, as illustrated in Figure 7b. A comparative analysis of (a) and (b) reveals that the shaded area in Figure 7a, representing wear traces, is concentrated in a specific region, which is primarily attributed to uneven stress distribution and non-uniform contact. In contrast, the shaded area in Figure 7b is more uniform and shallower, indicating that the optimized rotor experiences more uniform loading and improved contact uniformity. This comparison demonstrates the effectiveness of the optimization.

## 5. Conclusions

In this study, we proposed an optimization method that integrates the Kriging response surface model with a multi-objective genetic algorithm (MOGA) to address the design challenges of the flexible rotor in a traveling wave rotary ultrasonic motor (TRUM). Based on the existing TRUM60 model, we introduced a novel rotor design that is compatible with the improved stator. By focusing on the contact surface between the stator and rotor, an objective function was established and optimized using Latin hypercube sampling for Kriging model construction, followed by MOGA optimization. The optimization process resulted in a significant reduction in the objective function value from 0.631 to 0.036, while the maximum contact stress on the rotor’s inner ring decreased from 32.77 MPa to 9.96 MPa. Experimental results validated the reliability of the proposed design. The design of the flexible rotor for ultrasonic motors demonstrates significant improvements in addressing the uneven contact between the stator and rotor. This work highlights the effectiveness of combining advanced modeling techniques with optimization algorithms for complex motor design challenges. The findings of this study offer valuable insights for future research and development in the field of ultrasonic motors, particularly in improving contact uniformity and minimizing stress-related wear. But the current structural design is limited to the TRUM60-type ultrasonic motor and does not explore the applicability to other motor models. Additionally, this study lacks investigation into the microscopic contact behavior under ultrasonic frequencies. Future research will focus on microscopic analysis to address this gap.

## Figures and Tables

**Table 1:** Initial size and design space of the rotor.

Optimization Parameters	Initial Dimensions (mm)	Design Space (mm)
T1	1	[0.9, 1.1]
T2	2.5	[2.25, 2.75]
T3	2.5	[2.25, 2.75]
T4	1.8	[1.62, 1.98]
T5	16	[14.4, 17.6]
T6	0.8	[0.72, 0.88]
T7	0.75	[0.675, 0.825]
T8	0.75	[0.675, 0.825]
R6	8	[7.2, 8.8]

**Table 2:** Fixed size parameters of the rotor.

Parameter Type	Initial Dimensions
R1 (mm)	31.5
R2 (mm)	30.5
R3 (mm)	22
R4 (mm)	21
R5 (mm)	5
R7 (mm)	31.5
T9 (mm)	0.53
σs (MPa)	265

**Table 3:** Specific parameters of materials.

	Material	Density (kg/m^3^)	Young’s Modulus (G Pa)	Poisson’s Ratio
Rotor	Aluminum alloy	2780	73	0.31
Friction layer	Polytetrafluoroethylene	2100	1.2	0.45

**Table 4:** Parameter setting using MOGA.

Attribute	Value
Projected number of assessments	43,200
Initial sample size	9000
The number of samples per iteration	1800
Maximum allowable Pareto percentage	70
Convergence stability percentage	2
Maximum number of iterations	20
Mutation probability	0.01
Crossover probability	0.98
Maximum number of candidates	3

**Table 5:** Three groups of candidates.

	Candidate 1	Candidate 2	Candidate 3
T1	1.10	1.10	1.10
T2	2.65	2.60	2.62
T3	2.75	2.75	2.75
T4	1.64	1.60	1.70
T5	17.60	17.58	17.57
T6	0.79	0.78	0.80
T7	0.75	0.70	0.74
T8	0.82	0.82	0.82
R6	8.65	8.5	8.75

**Table 6:** The output values of the optimization objective function for the candidate and initial points.

	Candidate 1	Candidate 2	Candidate 3	Initial Point
M1	0.036	0.038	0.039	0.631
M2	9.96 MPa	9.98 MPa	10.0 MPa	32.77 MPa

## Data Availability

The data presented in this study are available on request from the corresponding author.
